# Evaluation of Somatic Mutations in Solid Metastatic Pan-Cancer Patients

**DOI:** 10.3390/cancers13112776

**Published:** 2021-06-03

**Authors:** Moom R. Roosan, Isa Mambetsariev, Rebecca Pharaon, Jeremy Fricke, Angel R. Baroz, Joseph Chao, Chen Chen, Mohd W. Nasser, Ramakanth Chirravuri-Venkata, Maneesh Jain, Lynette Smith, Susan E. Yost, Karen L. Reckamp, Raju Pillai, Leonidas Arvanitis, Michelle Afkhami, Edward W. Wang, Vincent Chung, Mihaela Cristea, Marwan Fakih, Marianna Koczywas, Erminia Massarelli, Joanne Mortimer, Yuan Yuan, Surinder K. Batra, Sumanta Pal, Ravi Salgia

**Affiliations:** 1 School of Pharmacy, Chapman University, Irvine, CA 92618, USA; roosan@chapman.edu; 2 Department of Medical Oncology & Therapeutics Research, City of Hope, Duarte, CA 91010, USA; Imambetsariev@coh.org (I.M.); rpharaon@coh.org (R.P.); jfricke@coh.org (J.F.); abaroz@coh.org (A.R.B.); jchao@coh.org (J.C.); suyost@coh.org (S.E.Y.); karen.reckamp@cshs.org (K.L.R.); edwang@coh.org (E.W.W.); vchung@coh.org (V.C.); mcristea@coh.org (M.C.); mfakih@coh.org (M.F.); mkoczywas@coh.org (M.K.); emassarelli@coh.org (E.M.); jmortimer@coh.org (J.M.); yuyuan@coh.org (Y.Y.); 3 Applied AI and Data Science, City of Hope, Duarte, CA 91010, USA; chechen@coh.org; 4 Department of Biochemistry and Molecular Biology, University of Nebraska Medical Center, Omaha, NE 68198, USA; wasim.nasser@unmc.edu (M.W.N.); r.chirravurivenkata@unmc.edu (R.C.-V.); mjain@unmc.edu (M.J.); sbatra@unmc.edu (S.K.B.); 5 Department of Biostatistics, University of Nebraska Medical Center, Omaha, NE 68198, USA; lmsmith@unmc.edu; 6 Cedars-Sinai Medical Center, Department of Medicine, Division of Medical Oncology, Los Angeles, CA 90048, USA; 7 Department of Pathology, City of Hope, Duarte, CA 91010, USA; rpillai@coh.org (R.P.); larvanitis@coh.org (L.A.); mafkhami@coh.org (M.A.)

**Keywords:** metastatic solid tumors, somatic mutations, overall survival, progression-free survival, molecular testing, targeted therapies

## Abstract

**Simple Summary:**

Cancer metastasis significantly contributes to cancer-related mortality. Our retrospective cohort study aimed to evaluate the mutational landscape of seven solid metastatic tumors and mutational effects on survival using a single molecular testing panel. Additionally, we assessed the treatments used in advanced cancer. We identified somatic mutations that were mutually exclusive in seven gene pairs. Among them, somatic mutations in *APC* and *CDKN2A* showed an opposite effect on overall survival (OS). Longer OS was associated with metastatic cases diagnosed post-2015. Progression-free survival was associated with the use of targeted treatments. Our results highlight complex interactions of mutational landscape with a single molecular test, time of metastatic diagnosis, and the impact of targeted therapy usage on survival using a pan-cancer cohort.

**Abstract:**

Metastasis continues to be the primary cause of all cancer-related deaths despite the recent advancements in cancer treatments. To evaluate the role of mutations in overall survival (OS) and treatment outcomes, we analyzed 957 metastatic patients with seven major cancer types who had available molecular testing results with a FoundationOne CDx^®^ panel. The most prevalent genes with somatic mutations were *TP53*, *KRAS*, *APC*, and *LRP1B*. In this analysis, these genes had mutation frequencies higher than in publicly available datasets. We identified that the somatic mutations were seven mutually exclusive gene pairs and an additional fifty-two co-occurring gene pairs. Mutations in the mutually exclusive gene pair *APC* and *CDKN2A* showed an opposite effect on the overall survival. However, patients with *CDKN2A* mutations showed significantly shorter OS (HR: 1.72, 95% CI: 1.34–2.21, *p* < 0.001) after adjusting for cancer type, age at diagnosis, and sex. Five-year post metastatic diagnosis survival analysis showed a significant improvement in OS (median survival 28 and 43 months in pre-2015 and post-2015 metastatic diagnosis, respectively, *p* = 0.00021) based on the year of metastatic diagnosis. Although the use of targeted therapies after metastatic diagnosis prolonged OS, the benefit was not statistically significant. However, longer five-year progression-free survival (PFS) was significantly associated with targeted therapy use (median 10.9 months (CI: 9.7–11.9 months) compared to 9.1 months (CI: 8.1–10.1 months) for non-targeted therapy, respectively, *p* = 0.0029). Our results provide a clinically relevant overview of the complex molecular landscape and survival mechanisms in metastatic solid cancers.

## 1. Introduction

Despite recent advances in targeted therapies, metastatic cancer continues to be one of the main causes of morbidity and mortality worldwide. Cancer results from the accumulation of mutations. The phenotypic features and survival outcomes of cancer can be linked to the mutational clone that enables it to proliferate, invade, and overcome its immune environment [1]. These mutational clones lead to heterogeneity, contributing to the challenges in selecting targeted treatment. A better understanding of the mutational landscape and personalized treatment is necessary. The advent of precision medicine has led to the identification of actionable driver mutations regardless of tumor site or origin [2]. The presence of this Darwinian dynamic drives cancer evolution in the process of natural selection of “driver” somatic mutations that give rise to persisting epigenetic changes responsible for proliferation and tumorigenesis [3,4]. The identification and the classification of these mutational drivers has unlocked the potential to therapeutically inhibit this process of perpetual autonomous expansion through targeted therapy [5,6,7,8,9].

The major NCI-MATCH study showed that molecular profiling was successful in 93% of cases, and an actionable alteration was identified in almost 40% of patients [2]. These results affirm the promise of precision medicine to transform clinical approaches from an organ-focused model to a comprehensive mutational signature model of cancer care. However, the application of targeted therapy across solid tumors has not been previously described in a real-world scenario. In this study, we have assessed and characterized the genomic and therapeutic landscape of a cohort of solid metastatic tumors at a single academic site. We performed a retrospective cohort analysis of 957 patients from seven major cancer groups who received next-generation sequencing (NGS) to investigate the relationship between alterations and overall survival (OS). The utilization of a single panel, FoundationOne CDx^®^, allowed us to independently evaluate the heterogeneity of our cohort, as well as to understand the alteration frequencies and their involvement in cancer diagnosis, progression, and OS.

## 2. Materials and Methods

### 2.1. Patient Characteristics

Patients (*N* = 1001) with solid tumors who underwent a commercial NGS molecular testing panel (Foundation Medicine, Cambridge, MA, USA) at City of Hope (COH) between 2010 and 2018 were evaluated. FoundationOne CDx^®^ assay is a clinically and analytically valid somatic mutation detection assay approved by FDA in November 2017 for all solid advanced tumors. Using their proprietary DNA extraction method from formalin-fixed paraffin-embedded (FFPE) tumor tissue samples, FoundationOne CDx^®^ can detect single nucleotide variants (SNVs), insertion-deletions (indels), and copy number alterations (CNAs) in 324 genes, as well as select rearrangements in 36 genes, microsatellite instability (MSI), and tumor mutational burden (TMB). Unlike hotspot tests, FoundationOne CDx^®^ offers comprehensive genomic profiling of the included genes. Therefore, the risk of selection bias of actionable mutation in this study is minimal with this assay for the solid cancer types analyzed. The COH patients with solid cancer with available FoundationOne CDx^®^ testing reports were eligible for evaluation in this study. The inclusion criteria for the current study include metastatic disease of solid cancers and availability of FoundationOne CDx^®^ test results after metastatic diagnosis. Cancer types that had less than 15 samples or samples that had an unknown date of metastatic disease diagnosis were excluded from the analysis. The majority of the specimens were collected from the primary sites as shown in Supplemental Table S1. Based on the inclusion-exclusion criteria, 957 patients were eligible for the study (Supplemental Figure S1). The clinical data collected from the electronic health record (EHR) included sex, age at diagnosis, date of metastatic diagnosis, race, stage, vital status at last contact, date of death/last contact, histology, cancer type, specimen type, date of molecular testing sample collection and results, first-line treatments, progression-free survival (PFS) and OS. The study was approved by the COH Institutional Review Board (IRB #18038) and the Department of Health and Human Services. The study was conducted according to the standards of Good Clinical Practice, the Declaration of Helsinki, and the US Department of Health and Human Services. All patients were evaluated for their written consent or exemption of consent according to requirements of the IRB and ethics committees, and all recorded data for this study were deidentified.

### 2.2. Statistical Analysis

Patient demographics and somatic mutations were summarized using descriptive statistics. Wilcoxon rank-sum test was used to compare the frequencies of mutations in two independent metastatic cohorts, specifically the COH pan-cancer and the University of Michigan 2017 metastatic cancer cohorts [10]. We evaluated mutation frequencies, co-occurrence, mutual exclusivity, and prognostic values. Cox regression models were used for univariate and multivariate analysis of OS endpoints with complete observations for clinical variables considered. For the mutation-based survival analysis, somatic mutations in 27 genes with 95 or more occurrences in the cohort were considered, and *p* ˂ 0.0017 (Bonferroni corrected for multiple testing) was considered to be significant for survival analyses using the Kaplan–Meier log-rank test. Mutations in >35 patient cases were considered for both co-occurrence and mutual exclusivity analysis. For multiple testing corrections, a false discovery rate (FDR) < 0.01 was considered to be statistically significant for analyses of co-occurrence and mutual exclusivity. For all other survival analyses, *p* < 0.05 was deemed to be significant. For pairwise OS comparison of APC and CDKN2A mutation statuses, Log-Rank test with Bonferroni adjusted *p* value was used for the significance test. Time to test was calculated in days between the date of metastatic diagnosis and FoundationOne CDx^®^ report date. Student t-test was used to evaluate times to test between pre-2015 and 2015 or later metastatic diagnosis. OS days were calculated from the date of metastatic diagnosis to the date of last follow-up, while PFS days were calculated from the date of metastatic diagnosis to the date of progression after first-line interventions. Data regarding PFS was obtained from the electronic medical record based on the primary oncologist’s notes and assessment of progression. We also compared our cohort to the mutation case frequencies from the Genomic Data Commons (GDC) and 500 adult patients with solid metastatic tumors [10,11].

The R packages used were ComplexHeatmap, GenVisR for heatmap analyses, discover for mutation co-occurrence and mutual exclusivity, and survival and survminer for OS analysis [12,13,14,15,16,17]. All analyses were performed using R version 3.6.2 [18].

## 3. Results

### 3.1. Clinical and Genomic Features

**Figure 1:**
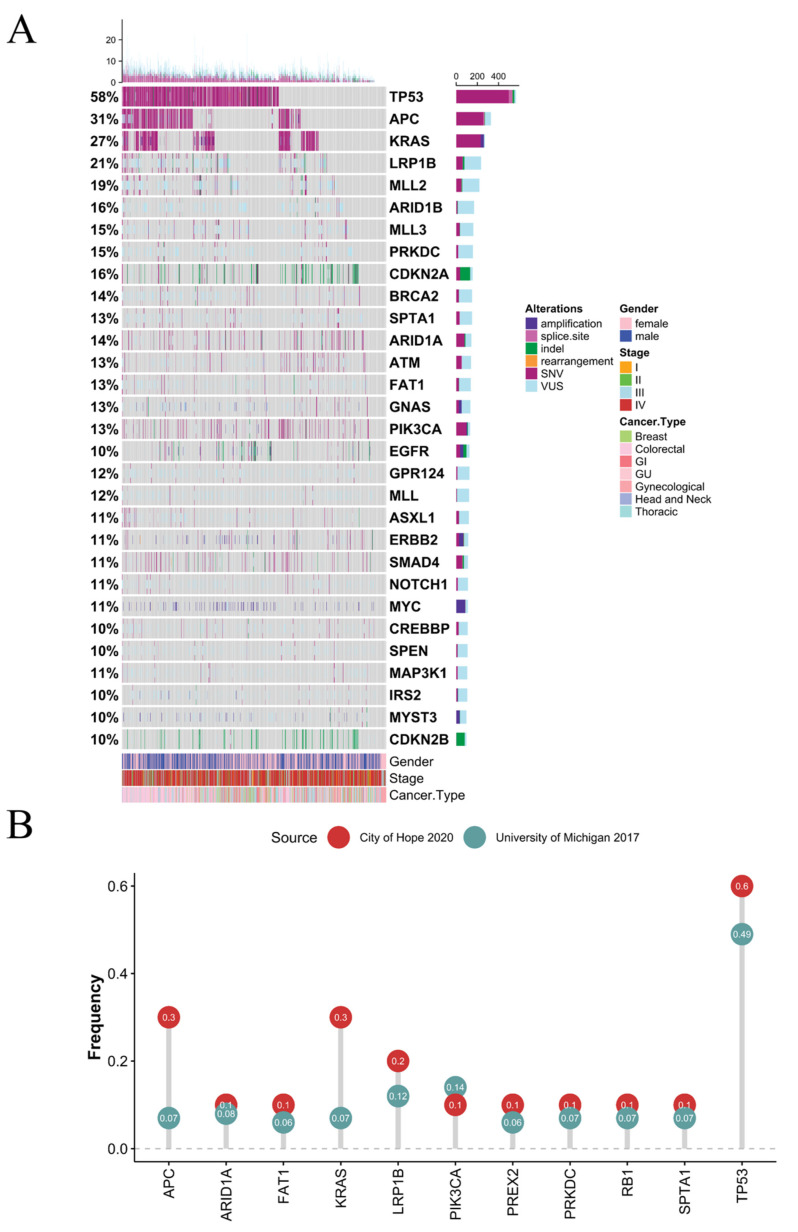
Mutation analysis of 957 patients treated at City of Hope. (**A**) Co-mutation plot of all somatic mutations with a frequency of 6% or more in 957 metastatic cancer patients. Row percentages represent the frequency of gene mutations in the cohort. Each of the column bars represents the number of mutations for each patient. SNV: single nucleotide variants; VUS: variant of unknown significance; GI: gastrointestinal; GU: genitourinary. (**B**) Comparison of most frequent mutations from the City of Hope 2020 and University of Michigan 2017 cohorts of solid metastatic cancer patients.

Of the 957 patients included in the analysis, 51.9% were female, and 69.7% were Caucasian. The median age at diagnosis was 60 years (IQR: 50–69 years) (Table 1). Seven major cancer diagnosis were included-breast (9.1%), colorectal (33.6%), gastrointestinal (GI; 12.9%), genitourinary (GU; 12.3%), gynecological (GYN; 6.2%), head and neck (H&N; 2%), and thoracic (23.9%) cancers. Colon adenocarcinoma (274, 28.6%) and lung adenocarcinoma (171, 17.9%) were the most common cancer subtypes in the cohort. A total of 390 (40.6%) patients harbored at least one targetable somatic mutation in actionable genes—AKT1, ATM, ALK, BRAF, BRCA1, BRCA2, EGFR, ERBB2, ESR1, FGF3, FGFR1, FGFR2, FGFR3, MET, NTRK1, NTRK2, NTRK3, PIK3CA, ROS1, and RET (NCCN colon, esophagus, head and neck, kidney, lung, ovary, prostate, urinary tract, and uterus guidelines; accessed on 13 December 2020) [19]. The most frequently altered gene in the COH pan-cancer cohort was TP53 (57.3%), followed by APC (31%) and KRAS (27%). In the COH cohort, TP53 alterations were the most prevalent in all cancer types except GU cancers (Figure 1, Supplemental Table S2). Colorectal cancer had the highest frequencies of TP53, APC, and KRAS mutations (76.4%, 73.9%, and 47.8%, respectively). H&N cancer had the highest frequencies of LRP1B (31.6%), and breast cancer had the highest frequency of PIK3CA and MYC (32.2% and 28.7%, respectively). Additionally, the COH pan-cancer cohort and the University of Michigan 2017 metastatic cancer cohort were significantly different within the top eleven common mutation frequencies (Figure 1B, Wilcoxon rank-sum test *p* 0.016). TP53, KRAS, and APC mutation frequencies were higher in the COH compared to the University of Michigan cohort [10]. In comparison with the GDC cancer types, TP53 was the most frequently altered mutation (35%). However, TP53 mutations occurred at a much higher rate in the COH cohort than the University of Michigan metastatic cohort (49%) and other primary tumor cohorts of TCGA (41%), TCGA 27.0 (36.6%), GENIE 8.1 (39%), and PCAWG (36.9%) [20,21,22,23]. The second most prevalent altered gene in our cohort was APC (31%), which was over 20% higher than the next highest dataset when compared to the University of Michigan metastatic cohort (0.07%) and mixed cohorts from TCGA (8%), TCGA 27.0 (8.1%), GENIE 8.1 (10.3%), and PCAWG (3.4%) [20,21,22,23]. The next most prevalent altered gene was KRAS (27%), which was 12% higher than TCGA (7%), TCGA 27.0 (9.8%), GENIE 8.1 (15.1%), and PCAWG (11.1%) [20,21,22,23].

### 3.2. Co-Occurrence and Mutual-Exclusivity in Pan-Cancer Cohort

**Figure 2:**
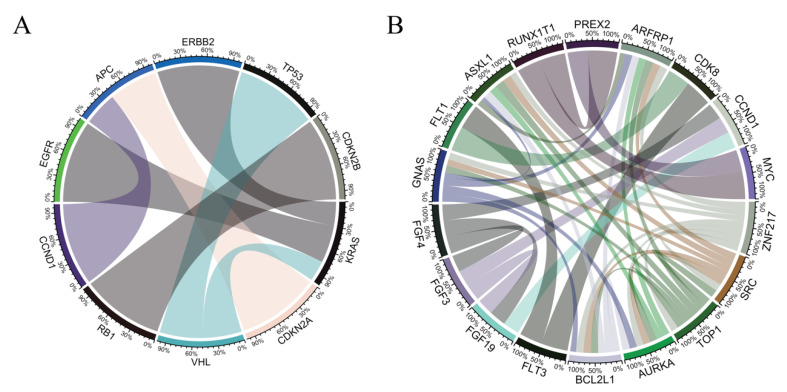
Mutually exclusive and co-occurring genes. Genes with more than 35 positive mutation cases were considered. Chord diagrams of (**A**) mutually exclusive, and (**B**) co-occurring gene pairs with a false discovery rate (FDR) less than 0.01. The arcs shown in the scaled chord-diagram between the pairs of genes represent the mutual exclusivity or co-occurrence. There were seven mutually exclusive gene pairs, of which KRAS was mutually exclusive with EGFR, ERBB2, and VHL. APC was mutually exclusive with CCND1 and CDKN2A. Thirty-one co-occurring gene pairs with more than one co-occurring gene pair were identified. ARFRP1, ASXL1, AURKA, BCL2L1, GNAS, SRC, TOP1, ZNF217 were most commonly co-occurring, and each co-occurred with seven other genes (FDR < 0.01).

There were 147 somatic mutations detected with more than 35 mutation cases within the cohort. Seven gene-pairs were found to be mutually exclusive and 51 gene-pairs to be co-occurring (Supplemental Table S3). Of the mutually exclusive pairs, KRAS was mutually exclusive with EGFR, ERBB2, and VHL. APC was mutually exclusive with CCDN1 and CDKN2A. Of these seven pairs, a groupwise mutual exclusivity analysis with eight genes (KRAS, EGFR, ERBB2, VHL, RB1, APC, CDKN2B, CCND1) were also mutually exclusive (*p* < 0.0001, Figure 2). Alternatively, ARFRP1, ASXL1, AURKA, BCL2L1, GNAS, SRC, TOP1, ZNF217 were the most commonly co-occurring, with each co-occurring with seven other genes (FDR < 0.01). Of the actionable genes, BRCA2, ERBB2, and MYC co-occurred with CDK8, CDK12, and RUNX1T1, respectively.

### 3.3. Survival Analysis

**Figure 3:**
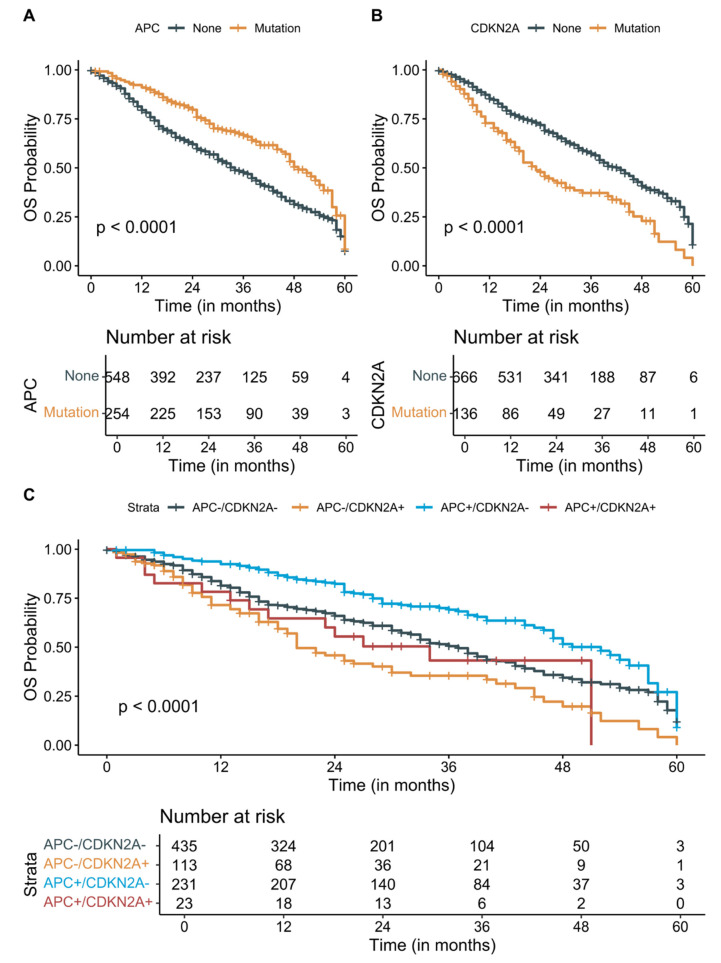
Patient survival analysis in the City of Hope Metastatic Dataset. Five-year Kaplan–Meier overall survival (OS) plots of patients with known (**A**) *APC*, (**B**) *CDKN2A*, and (**C**) both APC and *CDKN2A* mutations. Shorter OS was significantly associated with the presence of *CDKN2A* mutations, while *APC* mutations were significantly associated with longer OS. The OS benefit is observed in patients with *APC* and *CDKN2A* co-mutation compared to *CDKN2A* mutations only.

The COH metastatic solid cancer patients had a median PFS and OS of 10 months and 27 months, respectively. Out of 957 patients, 831 patients progressed despite the first-line interventions with pharmacotherapy, surgery, or radiation and 44 patients (21 colorectal, 4 GI, 7 GU, 2 gynecological, 10 thoracic cancer patients) did not progress. The remaining 82 patients had an unknown status of progression. The presence of APC (HR: 0.68, 95% CI: 0.54–0.85, *p* < 0.0001) and CDKN2A (HR: 2, 95% CI: 1.6–2.6, *p* < 0.0001) was significant predictors of the longer and shorter OS, respectively. However, only CDKN2A status remained significant (HR: 1.72, 95% CI: 1.34–2.21, *p* < 0.001, Supplemental Figure S2) after adjusting for age at metastatic diagnosis, gender, and cancer type in the multivariate Cox regression models. Due to the small number of patients with stable disease or remission, PFS was not significantly associated with any somatic mutation status. Patients with APC+/CDKN2A- metastatic cancer survived significantly longer than the patients who are APC-/CDKN2A+ (median survival 52 months compared to 20 months, *p* < 0.0001, Figure 3). Although APC and CDKN2A co-mutations are rare and have contrasting effects on OS, patients with APC mutation showed statistically nonsignificant longer OS in the presence of CDKN2A (median survival 34 months with APC+/CDKN2A+ mutations compared to 20 months with APC-/CDKN2A+ mutations).

### 3.4. Effect of Molecular Testing and First-Line Treatments

The median time from initial metastatic diagnosis to molecular testing was 265 days (IQR: 75–639 days) with a significantly accelerated time to testing since 2015 (mean 200.9 days compared to 890.6 days prior to 2015, *p* < 0.0001). The median time to reporting from sample collection was 115 days (IQR 41–401 days) and was significantly associated with time to testing (Pearson’s correlation: 0.46; *p* < 0.0001). Five-year post metastatic diagnosis survival analysis showed a significant improvement in OS (median survival 28 and 43 months in pre-2015 and post-2015 metastatic diagnosis, respectively, *p* 0.00021) by the year of metastatic diagnosis potentially due to faster time to testing and availability of targeted therapy based on the test results (Supplemental Figure S3).

**Figure 4:**
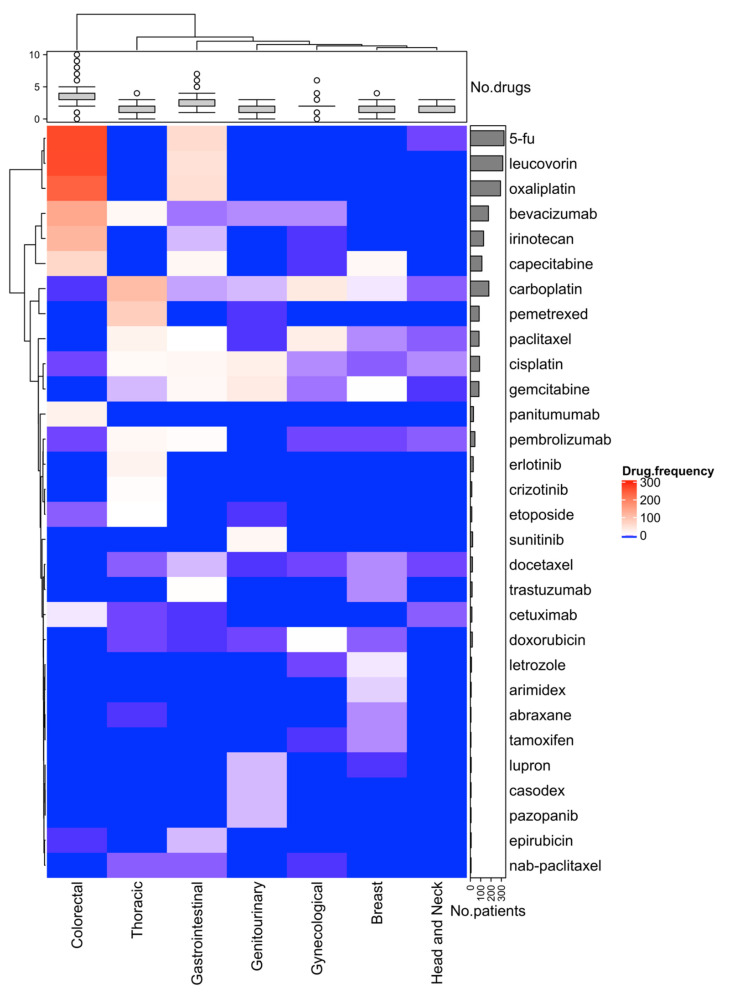
Frequency of the first-line drug usage for the cohort (*N* = 957 patients) by cancer type (**bottom**), the proportion of patients in each cancer type treated with each therapy (indicated by color on heat map), and the number of patients who received each treatment (**right**). The 30 most frequently administered first-line anticancer drugs are displayed. Top row box plots represent the median, upper, and lower quartiles of the number of drugs used in each cancer type, and whiskers represent the limits of the distribution.

**Figure 5:**
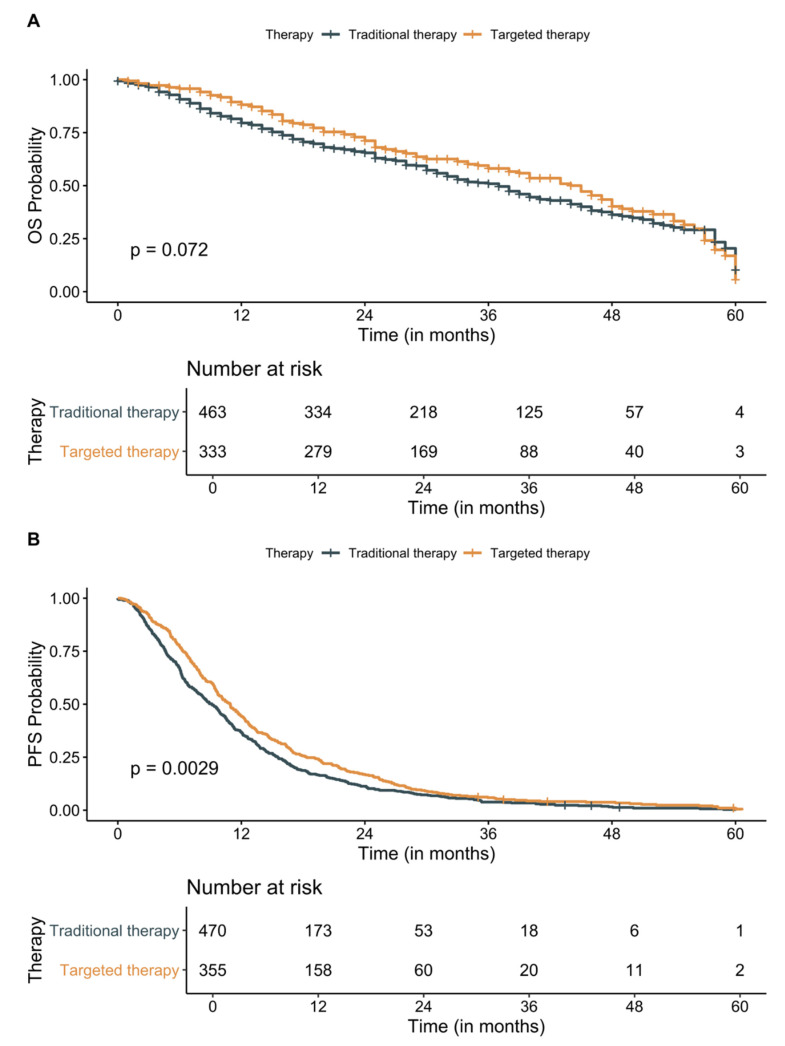
Kaplan–Meier 5-year (**A**) overall survival (OS) and (**B**) progression-free survival (PFS) analysis based on targeted versus traditional therapy use in solid pan-caner metastatic patients. Patients who received targeted therapies as the first-line treatment had improved but not statistically significant improvement in OS compared to patients who did not (median survival 45 months compared to 37 months for patients who received traditional therapies, *p* = 0.072). However, PFS was significantly improved in patients receiving targeted therapies (median PFS 9.1 months [CI: 8.1–10.1 months] compared to 10.9 months [CI: 9.7–11.9 months] in patients receiving traditional therapies, *p* = 0.0029). CI: confidence interval.

First-line treatment information was available for 941 (98.3%) patients, with 387 (40.4%) patients receiving targeted therapies as a first-line treatment after metastatic diagnosis. Of the known first-line treatments received by these patients, 5-FU, leucovorin, oxaliplatin, bevacizumab, irinotecan, capecitabine, and carboplatin were among the most frequently used chemotherapies across all seven cancer types (Figure 4). These drugs were most commonly used in colorectal and GI cancers. Carboplatin and pemetrexed regimen were most frequently administered in thoracic cancer. Although patients who received a targeted therapy as a first-line treatment after metastatic diagnosis tended to have a longer 5-year OS (45 months compared to 37 months for patients who received traditional therapies), the survival difference was not statistically significant (*p* = 0.072, Figure 5A). However, PFS was significantly longer for patients who received targeted therapies compared to patients who received traditional therapies such as chemotherapies, surgery, or radiation (median PFS 10.9 months [CI: 9.7–11.9 months] compared to 9.1 months [CI: 8.1–10.1 months], respectively, *p* 0.0029, Figure 5B). Use of targeted therapies as first-line treatment remained significantly associated with PFS (HR 0.81; 95% CI: 0.69–0.94; *p* 0.007) after adjusting for patient’s cancer type, age at diagnosis, and sex.

## 4. Discussion

Treatment of metastatic cancer continues to be challenging, resulting in high mortality among all cancer patients [24]. Here, we analyzed 957 metastatic solid cancer patients treated at the COH. In comparison with other published pan-cancer datasets, including The Cancer Genome Atlas (TCGA), the Pan-Cancer Analysis of Whole Genomes (PCAWG), and the recent data releases for the Genomics Evidence Neoplasia Information Exchange (GENIE) 8.1 and TCGA 27.0, there was significant variation in top somatic mutation frequencies of TP53, APC, KRAS, and LRP1B genes [20,21,22,23]. One possible explanation for the differences in frequency is the metastatic status of our cohort compared to the mixed cancer patient population from TCGA 27.0 and GENIE. Further, there were only 957 patients in our COH cohort, while the most recent data releases for TCGA 27.0 and GENIE 8.1 were for 84,392 and 95,918 primary tumor samples, respectively [21,22,25]. Also, we analyzed breast, colorectal, gastrointestinal, genitourinary, gynecological, head and neck, and thoracic major cancer types. Colorectal (322, 33.6%) and thoracic (229, 23.9%) accounted for 57.5% of all cancer diagnosis in our cohort, and TP53, APC, KRAS, and LRP1B mutation rates are higher in colorectal cancers and non-small cell lung cancers (NSCLC) [26,27,28,29]. APC gene mutations are known to be associated with chromosomal instability, however, similar to our findings several studies have identified that mutated APC was associated with improved survival as compared to wild-type APC in colorectal cancer [30,31,32,33,34].

Mutual exclusivity and co-occurrence among oncogenic driver mutations warrant careful discussion when considering targeted therapies and treatment plans. While biology drives mutual exclusivity, most co-occurrences are most likely by chance [14]. Previous studies have reported the prevalence of concomitant driver mutations among the different patient populations. In our study, the seven mutually exclusive gene pairs with FDR < 0.01 are KRAS/EGFR, CDKN2A/APC, KRAS/ERBB2, VHL/TP53, RB1/CDKN2B, VHL/KRAS, and CCND1/APC. A well-known pair of genes that validates our mutual exclusion data is KRAS with mutated EGFR loci, most commonly seen in lung cancer [35]. The EGFR family of receptors EGFR and HER2 (ERBB2) act as upstream regulators of KRAS in the Ras/Raf/MEK/ERK pathway. Concurrent mutations in EGFR or ERBB2 and KRAS are not selected for and may be considered redundant in tumor cells, thus selective pressures may account for the mutual exclusivity of EGFR/ERBB2 and KRAS [36,37,38]. However, recent publications have shown small cohorts of patients that harbor the rare EGFR/KRAS co-mutations. Zhuang et al. reported a comprehensive study analyzing concomitant driver gene mutations in 3774 NSCLC Chinese patients harboring EGFR, ALK, ROS1, KRAS, and BRAF alterations. Only 63 patients (1.7%) harbored mutations in two or three of these genes and among these patients, EGFR/KRAS was the most frequent co-alteration (31.7%) [39]. Pesek et al. reported four clinical cases of NSCLC patients with confirmed co-mutations in four of the five clinical cases presented [40]. Additionally, our analysis confirmed that the presence of TP53 mutations was mutually exclusive with VHL mutations. Previously, it was found that mutual exclusivity among the mutations depends on different histological subtypes in renal cell carcinomas (RCCs) [41]. Szymańska et al. reported 77% of clear-cell carcinomas from a multi-center case-control study harbor VHL mutations; in contrast, only 22% of RCCs of other histological types harbor VHL mutations [42]. Upon stratification, clear cell tumors showed mutual exclusivity among TP53 and VHL mutations, whereas non-clear cell tumors had TP53 mutations in the absence of VHL mutations.

We reported the following actionable gene pairs as co-occurring—ERBB2/CDK12, BRCA2/CDK8, and MYC/RUNX1T1 with additional gene pairs, including PREX2/RUNX1T1 and STK11/KEAP1. Somatic mutations that co-occur are common across various cancer types. For example, CDK12 is an essential transcription-associated CDK responsible for DNA damage response and is often found in the ERBB2 amplicon [43,44,45]. Capra et al. reported that CDK12 gene amplification can often co-occur with ERBB2 amplification and may act as an oncogenic driver in HER2-positive breast cancer [46,47,48]. Wilson et al. reported similar co-occurring gene pair mutations among breast cancer subtypes (HER2+, hormone receptor positive [HR+], and triple-negative breast cancer [TNBC]) [49]. Interestingly, the commonly occurring gene pairs MYC/RUNX1T1, PREX2/RUNX1T1, PRKDC/PREX2, and MYC/PREX2 in our study are coamplified genes on the same loci, within the 8q loci, and reported by Wilson et al. in 13% of patients with HR+, HER2- disease and 19% with TNBC [49]. Lastly, our analysis showed STK11/KEAP1 co-occurring mutations (FDR < 0.01). Concurrent mutations in STK11/KEAP1 act as a major driver in primary resistance to PD1 blockade in KRAS-mutated lung adenocarcinoma [50]. Papillon-Cavanagh et al. investigated the utility of STK11/KEAP1 mutations as predictive biomarkers in patients diagnosed with stage IIIB, IIIC, IVA, or IVB non-squamous NSCLC. It was determined that the co-occurring mutations were associated with poor prognosis in both anti-PD-1/PD-L1 treated and chemotherapy-treated populations [51].

Rapid advancements in NGS technology have allowed physicians to understand in real-time the genetic makeup of a patient’s tumor and track these alterations over time [52,53,54]. Groisberg et al. reported the identification of an oncogenic mutation in at least 92% of patients [55]. In a more comprehensive analysis of 10,000 patients, Zehir et al. reported 91% successful sequencing and identified 36.7% of patients with at least one actionable alteration [56]. These rates of actionable alterations were previously estimated to be lower, but recent studies show that actionability in solid tumors can vary between 35–80% [57,58,59,60,61]. This has been compounded by the recent results from the NCI-match study that sequenced 93% of 5954 patients and identified actionable alterations in 37.8% of cases [2]. Our results suggest a similar frequency to other NGS-guided studies that match targeted therapy based on the identification of at least one actionable alteration [62,63]. The advent of novel therapeutics, including new NTRK, RAS, RET, and MET inhibitors has transformed the clinical trial landscape and presented more therapeutic options to patients than ever before [64,65,66,67]. These advancements in genomic sequencing and trial accrual validate precision medicine to deliver improved outcomes in all solid tumors.

It is worth noting that our study has several limitations. First, this is a retrospective cohort study using seven solid metastatic tumor patients from a single center. Therefore, the study results may not be generalizable to other metastatic cohorts. Second, survival may depend on various factors beyond the age of diagnosis, sex, cancer type, and treatments used. Also, treatment options are different depending on cancer types, and the efficacy of treatments may vary significantly based on how soon in the disease progression they were initiated. Therefore, our survival assessment was oversimplified. Third, we did not capture clonal heterogeneity and evaluate the effect of intratumor heterogeneity on OS. Future studies need to address these limitations in the evaluation of somatic mutations in solid metastatic pan-cancer patients.

## 5. Conclusions

The promise of precision medicine relies on identifying mutations in patients and developing novel therapeutics for these drug targets. Beyond actionable mutations, gene expression and epigenetics data may further elucidate critical pathway activation and identify potential targeted therapies [68,69]. Although gene expression and epigenetics data are not routinely implemented in clinical decision-making, using these in collaboration with mutation data will shed light on the selection of optimal targeted therapies and potentially avoid resistance development. Targeted therapies are becoming more common and the efficacy of these agents has been proven in several solid tumors [67,70,71,72,73,74,75,76]. These agents provide a measurable outcome advantage compared to other therapeutic options, including chemotherapy and immunotherapy—with the longest median OS of 7 years in patients with ALK-mutated metastatic NSCLC, an achievement unmatched by any other therapeutic option [77]. However, the primary barrier to targeted therapeutics remains acquired resistance due to evolutionary competition between resistant and sensitive subclones, which leads to survival of persistent subclones that alter tumor behavior and outcomes [78,79,80,81]. Our results showed the application of targeted therapies improved PFS, potentially owing to early testing and availability of increasing number of targeted therapies. Combinatory targeted therapeutic options are a potential non-toxic avenue for combatting this resistance, and several early and late-stage clinical trials, including NCI #10327, NCI #9466, and NCT03944772, are currently underway to understand their efficacy. The promise of precision medicine is quickly transforming into the reality of precision therapeutics with the discovery of effective KRAS-inhibitors and other drug targets that were previously thought to be undruggable [65,71]. Therefore, the outcomes of patients with solid tumors rely on the successful inhibition of oncogenic mutational signatures that drive disease progression by utilizing informed targeted therapy.

## Figures and Tables

**Table 1:** Baseline characteristics of 957 patients with seven metastatic solid cancers. IQR: interquartile range; MSI: microsatellite instability.

Characteristic	Pan-Cancer (*n* = 957)	Breast (*n* = 87)	Colorectal (*n* = 322)	Gastrointestinal (*n* = 123)	Genitourinary (*n* = 118)	Gynecological (*n* = 59)	Head and Neck (*n* = 19)	Thoracic (*n* = 229)
Age at diagnosis, median (IQR)	60 (50–69)	50 (42–56)	56 (48–66)	63 (49–70)	67 (60–73)	61 (51.5–71)	61 (50.5–65.5)	64 (54–72)
Race, *n* (%)								
White	667 (69.7)	50 (57.5)	230 (71.4)	74 (60.2)	98 (83.1)	47 (79.7)	17 (89.5)	151 (65.9)
Asian	194 (20.3)	18 (20.7)	60 (18.6)	35 (28.5)	13 (11)	8 (13.6)	1 (5.3)	59 (25.9)
African American	47 (4.9)	12 (13.8)	15 (4.7)	4 (3.3)	2 (1.7)	2 (3.4)	1 (5.3)	11 (4.8)
Other	18 (1.9)	4 (4.6)	7 (2.1)	7 (5.7)	3 (2.5)	2 (3.7)	0 (0)	4 (1.7)
Unknown	22 (2.3)	3 (3.5)	10 (3.1)	3 (2.4)	2 (1.6)	0 (0)	0(0)	4 (1.7)
Ethnicity, *n* (%)								
Hispanic or Latino	193 (20.2)	24 (27.6)	74 (23)	37 (30.1)	10 (8.5)	16 (27.1)	3 (15.8)	29 (12.7)
Not Hispanic or Latino	752 (78.6)	61(70.1)	244 (75.8)	83 (67.5)	108 (91.5)	43 (72.9)	16 (84.2)	197 (86)
Unknown or not disclosed	12 (1.2)	2(2.3)	4 (1.2)	3 (2.4)	0 (0)	0 (0)	0 (0)	3 (1.3)
Sex, *n* (%)								
Female	497 (51.9)	87 (100)	138 (42.9)	54 (43.9)	27 (22.9)	59 (100)	8 (42.1)	124 (54.1)
Male	460 (48.1)	0 (0)	184 (57.1)	69 (56.1)	91 (77.1)	0 (0)	11 (57.9)	105 (45.9)
Tumor Burden, *n* (%)								
Low	204 (21.2)	19 (21.8)	70 (21.7)	20 (16.3	16 (13.1)	21 (35.6)	10 (52.6)	48 (21.1)
Intermediate	115 (12)	6 (6.9)	37 (11.5)	17 (13.8)	8 (6.6)	10 (16.9)	3 (15.8)	34 (14.9)
High	17 (1.8)	0 (0)	2 (0.6)	1 (0.8)	1 (0.8)	1 (1.7)	2 (10.5)	10 (4.4)
Unknown	624 (65)	62 (71.2)	221 (66.1)	85 (69.1)	97 (79.5)	27 (45.8)	4 (21.1)	136 (59.6)
Stage at initial diagnosis, *n* (%)								
I	41 (4.3)	16 (18.4)	9 (2.8)	0 (0)	0 (0)	5 (8.5)	1 (5.3)	10 (4.4)
II	81 (8.5)	32 (36.8)	29 (9)	2 (1.6)	0 (0)	4 (6.8)	3 (15.8)	11 (4.8)
III	134 (14)	23 (26.4)	62 (19.3)	3 (2.4)	4 (3.9)	23 (39)	0 (0)	19 (8.3)
IV	701 (73.3)	16 (18.4)	222 (68.9)	118 (95.9)	114 (96.6)	27 (45.8)	15 (78.9)	189 (82.5)
MSI status, *n* (%)								
High	8 (0.8)	0 (0)	2 (0.6)	2 (1.6)	0 (0)	1 (1.7)	1 (5.3)	2 (0.9)
Stable	351 (36.7)	26 (29.9)	116 (36)	39 (31.7)	25 (21.2)	32 (54.8)	15 (78.9)	98 (42.8)
Unknown	598 (62.5)	60 (70.1)	204 (63.4)	82 (66.7)	93 (78.8)	26 (44.1)	3 (15.8)	129 (56.3)

## Data Availability

All of the pertinent data and codes for the study is available at https://github.com/mumtahena/COH_metastatic_solid (accessed on 4 January 2020).

## Supplementary Materials

The following are available online at https://www.mdpi.com/article/10.3390/cancers13112776/s1: Figure S1: Study Flow Diagram Showing Metastatic Pan-cancer Patient Selection for Analysis. A total of 1001 patients with available FoundationOne CDx® test results were evaluated. Three patients with unknown cancer types, one patient with skin cancer and forty patients without test results after metastatic diagnosis were excluded, resulting in 957 eligible patients for the study analysis. Figure S2: Forest plot of variables analyzed for overall survival. Hazard ratios of each variable along with the p-value are shown. Mutations in CDKN2A gene was associated with significantly worse survival after adjusting for age at diagnosis, sex, APC mutations and cancer type. Figure S3: Kaplan-Meier overall survival analysis in solid pan-cancer metastatic patients five year after metastatic diagnosis. Patients who had the metastatic diagnosis before 2015 had significantly shorter overall survival compared to those diagnosed in 2015 or later. Table S1: Summary of the specimen site classifications for the various cancer subgroups. Table S2: Prevalence in cases (%) of the top thirty mutated genes across seven cancer types. Table S3: List of all mutually exclusive and co-occurring gene pairs with false discovery rate (FDR) < 0.01.
