# Supplementary material for: Evaluation of Somatic Mutations in Solid Metastatic Pan-Cancer Patients
Source: Cancers (Basel). 2021 Jun 3;13(11):2776. doi: 10.3390/cancers13112776 (PMC8199748; doi:10.3390/cancers13112776)
Supplement: Supplementary file 1 [file cancers-13-02776-s001.zip › cancers-1205106-supplementary.pdf]

Supplemental Materials:

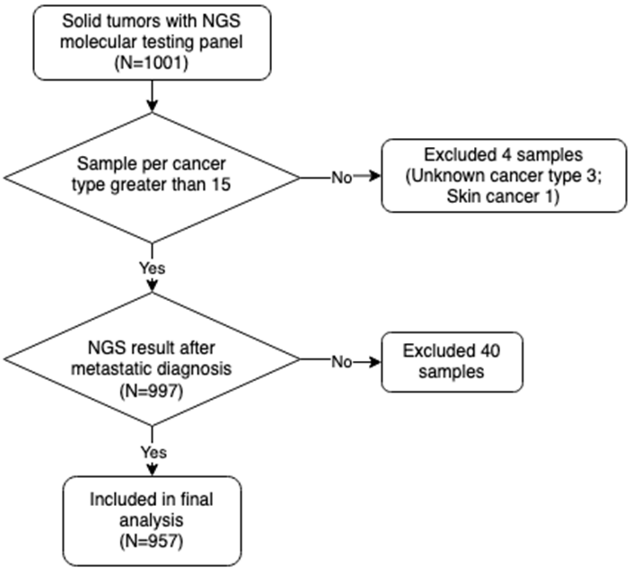

Figure S1: Study Flow Diagram Showing Metastatic Pan-cancer Patient Selection for Analysis. A total of 1001 patients with available FoundationOne CDx® test results were evaluated. Three patients with unknown cancer types, one patient with skin cancer and forty patients without test results after metastatic diagnosis were excluded, resulting in 957 eligible patients for the study analysis.

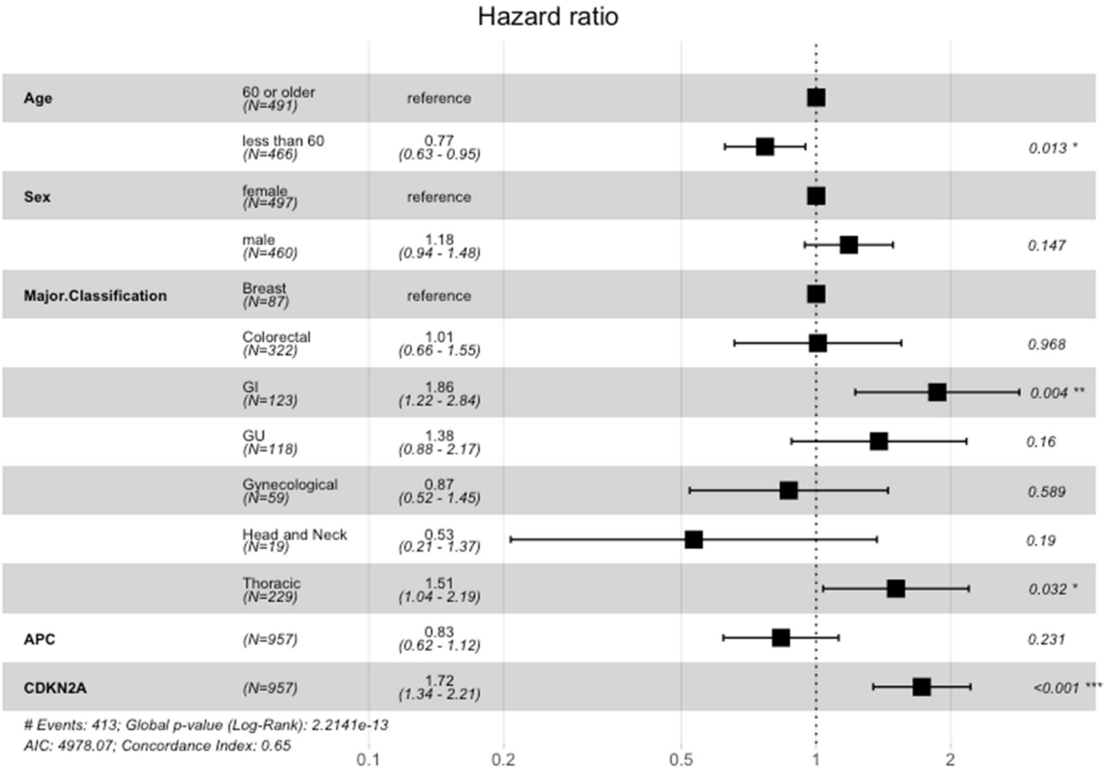

Figure S2: Forest plot of variables analyzed for overall survival. Hazard ratios of each variable along with the p-value are shown. Mutations in CDKN2A gene was associated

with significantly worse survival after adjusting for age at diagnosis, sex, APC mutations and cancer type.

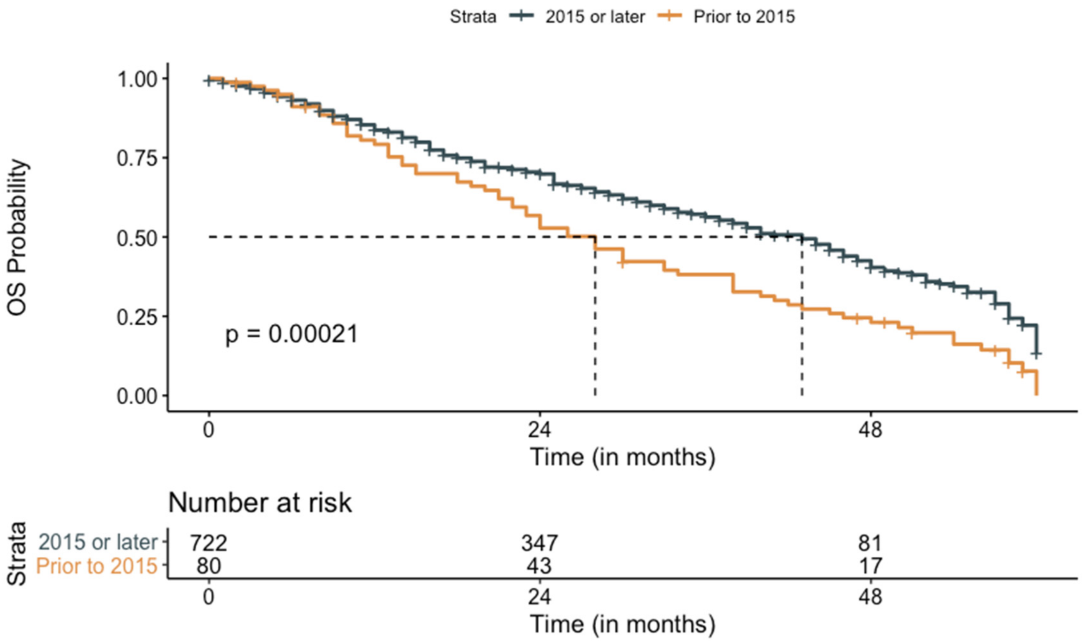

Figure S3: Kaplan-Meier overall survival analysis in solid pan-cancer metastatic patients five year after metastatic diagnosis. Patients who had the metastatic diagnosis before 2015 had significantly shorter overall survival compared to those diagnosed in 2015 or later.

Table S1. Summary of the specimen site classifications for the various cancer subgroups.

Cancer	Primary	Metastatic
Breast Cancer	27	60
Colorectal Cancer	192	139
GI Cancer	79	44
Thoracic cancer	166	83
Genitourinary Cancer	75	51
Gynecological Cancer	20	42
Head and Neck Cancer	7	12

Table S2: Prevalence in cases (%) of the top thirty mutated genes across seven cancer types.

	Breast (N=87)	Colorectal (N=322)	GI (N=123)	GU (N=118)	Gynecological (N=59)	Head and Neck (N=19)	Thoracic (N=229)
**TP53**	60.9	76.4	57.7	29.7	35.6	47.4	52
**APC**	9.2	73.9	6.5	12.7	3.4	10.5	10.5
**KRAS**	4.6	47.8	22	3.4	10.2	10.5	26.6
**LRP1B**	14.9	17.7	21.1	20.3	15.3	31.6	27.1
**MLL2**	18.4	16.5	17.9	33.1	16.9	21.1	16.2
**ARID1B**	14.9	15.2	17.1	18.6	13.6	21.1	17
**CDKN2A**	10.3	3.7	24.4	28.8	5.1	26.3	24.5
**MLL3**	16.1	15.8	13	14.4	18.6	15.8	13.5
**PRKDC**	23	14.9	13.8	16.1	13.6	10.5	10.9
**ARID1A**	11.5	9.6	25.2	16.1	13.6	15.8	15.3
**BRCA2**	16.1	17.4	12.2	7.6	15.3	15.8	13.1
**ATM**	14.9	12.4	14.6	16.1	8.5	21.1	12.7
**SPTA1**	18.4	11.8	7.3	15.3	13.6	15.8	15.3
**FAT1**	5.7	15.5	15.4	11.9	5.1	31.6	12.2
**PIK3CA**	32.2	13.7	7.3	12.7	20.3	10.5	6.6
**GNAS**	13.8	19.6	15.4	4.2	5.1	0	8.3
**MLL**	14.9	12.1	13.8	11	8.5	10.5	12.2
**GPR124**	21.8	10.2	11.4	9.3	10.2	15.8	12.2
**ASXL1**	8	17.7	8.1	8.5	8.5	10.5	8.3
**MYC**	28.7	10.9	8.1	10.2	6.8	0	9.6
**SMAD4**	3.4	20.5	17.9	0.8	1.7	0	6.1
**NOTCH1**	17.2	10.9	9.8	11.9	3.4	10.5	10
**ERBB2**	18.4	6.5	14.6	12.7	8.5	0	11.8
**MAP3K1**	16.1	9.6	8.9	11	11.9	26.3	8.7
**CREBBP**	14.9	9.6	7.3	11.9	6.8	5.3	10.9
**EGFR**	4.6	4	4.9	6.8	0	5.3	28.4
**SPEN**	13.8	12.7	12.2	6.8	5.1	10.5	7
**CDKN2B**	10.3	2.5	13.8	18.6	3.4	5.3	15.3
**IRS2**	10.3	11.8	13.8	4.2	5.1	10.5	8.7
**MYST3**	19.5	9.3	6.5	6.8	15.3	5.3	8.3

Table S3: List of all mutually exclusive and co-occurring gene pairs with false discovery rate (FDR) <0.01.

Gene 1	Gene 2	p-value	False Discovery Rate (FDR)	Mutually exclusive/co-occurring
KRAS	EGFR	3.86E-09	9.51E-07	Mutually exclusive
CDKN2A	APC	7.06E-07	0.00016613	Mutually exclusive
KRAS	ERBB2	4.46E-06	0.00093275	Mutually exclusive
VHL	TP53	7.06E-06	0.00122194	Mutually exclusive
RB1	CDKN2B	1.06E-05	0.00159381	Mutually exclusive
VHL	KRAS	2.25E-05	0.00321852	Mutually exclusive
CCND1	APC	6.35E-05	0.00957094	Mutually exclusive
ZNF217	AURKA	-2.69E-14	0	Co-occurring
SRC	AURKA	-2.02E-14	0	Co-occurring
ZNF217	SRC	-1.71E-14	0	Co-occurring
CDKN2B	CDKN2A	-1.33E-14	0	Co-occurring
TOP1	AURKA	-1.07E-14	0	Co-occurring
TOP1	BCL2L1	-9.33E-15	0	Co-occurring
AURKA	ARFRP1	-8.66E-15	0	Co-occurring
BCL2L1	ARFRP1	-8.44E-15	0	Co-occurring
FLT3	CDK8	-6.88E-15	0	Co-occurring
ZNF217	TOP1	-6.00E-15	0	Co-occurring
FGF19	CCND1	-4.88E-15	0	Co-occurring
FGF3	CCND1	-4.88E-15	0	Co-occurring
SRC	BCL2L1	-3.33E-15	0	Co-occurring
ZNF217	ARFRP1	-2.44E-15	0	Co-occurring
FGF4	FGF19	-2.22E-15	0	Co-occurring
FGF4	FGF3	-2.22E-15	0	Co-occurring
SRC	ASXL1	-2.00E-15	0	Co-occurring
TOP1	ARFRP1	-1.11E-15	0	Co-occurring
TOP1	SRC	-6.66E-16	0	Co-occurring
TOP1	ASXL1	2.22E-16	5.35E-14	Co-occurring
SRC	ARFRP1	4.11E-15	9.59E-13	Co-occurring
ZNF217	BCL2L1	5.11E-15	1.13E-12	Co-occurring
BCL2L1	AURKA	5.55E-15	1.13E-12	Co-occurring
FGF4	CCND1	5.55E-15	1.13E-12	Co-occurring
FGF3	FGF19	6.88E-15	1.37E-12	Co-occurring
GNAS	AURKA	1.09E-14	2.04E-12	Co-occurring
BCL2L1	ASXL1	1.87E-14	3.44E-12	Co-occurring
GNAS	ARFRP1	4.65E-14	8.42E-12	Co-occurring
AURKA	ASXL1	1.41E-13	2.44E-11	Co-occurring
FLT3	FLT1	1.74E-12	2.96E-10	Co-occurring

FLT1	CDK8	1.99E-12	3.25E-10	Co-occurring
GNAS	BCL2L1	4.27E-12	6.76E-10	Co-occurring
ZNF217	ASXL1	6.97E-12	1.09E-09	Co-occurring
ZNF217	GNAS	1.11E-11	1.71E-09	Co-occurring
TOP1	GNAS	2.12E-11	3.15E-09	Co-occurring
ASXL1	ARFRP1	2.31E-11	3.37E-09	Co-occurring
SRC	GNAS	1.49E-10	2.13E-08	Co-occurring
ZNF703	FGFR1	2.74E-08	4.00E-06	Co-occurring
RICTOR	IL7R	7.03E-08	1.02E-05	Co-occurring
ERBB2	CDK12	4.03E-07	5.80E-05	Co-occurring
GNAS	ASXL1	7.52E-07	0.00010611	Co-occurring
RUNX1T1	PREX2	9.99E-07	0.00013928	Co-occurring
ZNF703	GPR124	1.55E-06	0.00021131	Co-occurring
SDHA	IL7R	3.20E-06	0.0004283	Co-occurring
RUNX1T1	MYC	3.99E-06	0.00052653	Co-occurring
VHL	PBRM1	1.15E-05	0.00150192	Co-occurring
PRKDC	PREX2	1.81E-05	0.00233896	Co-occurring
STK11	KEAP1	3.04E-05	0.00393447	Co-occurring
MCL1	DDR2	4.24E-05	0.00538202	Co-occurring
PREX2	MYC	6.15E-05	0.00761615	Co-occurring
CDK8	BRCA2	7.23E-05	0.00876214	Co-occurring
